# Apple dwarfing rootstocks exhibit an imbalance in carbohydrate allocation and reduced cell growth and metabolism

**DOI:** 10.1038/hortres.2017.9

**Published:** 2017-04-05

**Authors:** Toshi M Foster, Peter A McAtee, Chethi N Waite, Helen L Boldingh, Tony K McGhie

**Affiliations:** 1 The New Zealand Institute for Plant and Food Research Limited, Palmerston North 4474, New Zealand; 2 The New Zealand Institute for Plant and Food Research Limited, Auckland 1142, New Zealand; 3 The New Zealand Institute for Plant and Food Research Limited, Hamilton 3240, New Zealand

## Abstract

Apple dwarfing rootstocks cause earlier shoot termination and reduced root and shoot mass. To identify physiological factors responsible for rootstock-induced growth restriction, we compared vascular-enriched gene expression between two dwarfing rootstocks (‘M27’ and ‘M9’) and the vigorous rootstock ‘M793’ using RNA sequencing and quantitative reverse transcriptase PCR. Differentially expressed genes common to both dwarfing rootstocks belonged to five main biological processes: (1) primary metabolism, (2) cell wall synthesis and modification, (3) secondary metabolism, (4) hormone signalling and response and (5) redox homeostasis. Genes promoting the biosynthesis of amino acids, lipids and cell walls were downregulated in dwarfing rootstocks, whereas genes promoting the breakdown of these compounds were upregulated. The only exception to this trend was the upregulation of starch synthesis genes in dwarfing rootstocks. Non-structural carbohydrate analysis demonstrated that starch concentrations in ‘M9’ roots, stems and grafted ‘Royal Gala’ (‘RG’) scions were double that of equivalent tissues from ‘RG’ homo-grafted trees (‘RG’/‘RG’). Fructose and glucose concentrations were much lower in all three tissues of the ‘RG’/‘M9’ trees. Together, these data indicate that dwarfing rootstocks are in a state of sugar depletion and reduced cellular activity despite having large starch reserves. Another significant finding was the over-accumulation of flavonoids and the downregulation of auxin influx transporters MdAUX1 and MdLAX2 in dwarfing rootstocks. We propose that both factors reduce polar auxin transport. The results of this study contribute novel information about the physiological state of dwarfing rootstocks.

## INTRODUCTION

Dwarfing rootstocks are widely used in commercial apple production to reduce scion vigour, allowing high-density plantings and increased yield index. Despite their utility and a century of research, the underlying mechanism of rootstock-induced dwarfing is still unknown. The effects of dwarfing rootstocks are apparent within the first year after grafting and include reduced root growth,^1–3^ a decrease in the length and node number of the primary axis,^4–6^ a decrease in the number and length of sylleptic shoots^7,8^ and/or an increase in the proportion of floral buds along the primary axis.^8–10^

The most widely used and best-characterised dwarfing apple rootstock is ‘Malling 9’ (‘M9’), which was first phenotyped in the early twentieth century.^11^ The ‘Malling’ series have been used to breed new rootstocks that confer a range of vigour control, including the strongly dwarfing ‘M27’. Rootstock-induced dwarfing is conferred by two major quantitative trait loci.^12–15^ Most of the known dwarfing rootstocks carry markers linked to one or both loci, suggesting that there is one source of dwarfing rootstocks and they reduce scion vigour by the same underlying physiological mechanism.^14,15^

Many of the hypotheses to explain how dwarfing rootstocks affect scion growth include altered levels, transport and signalling of hormones between scion and rootstock. Perhaps, the best-supported model proposes that dwarfing rootstocks reduce basipetal auxin transport, thereby limiting root growth and the amount of root-synthesised cytokinin supplied to the scion.^16^ Stem segments of ‘M9’ transport radiolabelled IAA (auxin) at a lower rate than that of vigorous genotypes.^17,18^ Levels of free IAA and Zeatin (cytokinin) in cambial sap are lower in ‘M9’ than vigorous rootstocks.^19^ Other studies have shown an inverse relationship between the rate of IAA diffusion and xylem concentration of cytokinin.^2^ Van Hooijdonk *et al.*^20^ demonstrated that application of the polar auxin inhibitor 1*-N*-naphthylphthalamic acid to the stem of an invigorating rootstock had the same effect on scion growth as that of the ‘M9’ rootstock. Application of cytokinin and 1*-N*-naphthylphthalamic acid restored the number of secondary shoots to levels typical of scions on vigorous rootstocks. Likewise, application of cytokinin to scions grafted onto ‘M9’ increased the number of secondary shoots.^20^ Other hormones may also have a role in rootstock-induced dwarfing. The concentration of gibberellic acid in the xylem is lower in ‘M9’ relative to vigorous rootstocks.^2,21^ Application of gibberellic acid to 1*-N*-naphthylphthalamic acid-treated vigorous rootstocks or scions on ‘M9’ increased node number of both the primary axis and secondary shoots.^20^ Abscisic acid (ABA) concentrations are higher in dwarfing than in vigorous rootstocks.^22,23^

Anatomical factors have also been implicated as causal factors in rootstock-induced dwarfing. The roots and stems of dwarfing rootstocks have a higher proportion of bark, which consists of phloem and cortex, and a reduction in the number and diameter of xylem cells relative to that of vigorous rootstocks.^24–26^ Vasculature at the graft junction between ‘M.9’ and the scion is disorganised, suggesting auxin accumulation in the region, but no evidence of graft incompatibility was found.^25,26^ Some researchers have suggested that a smaller root system and reduced amount of conducting tissues observed in dwarfing rootstocks were limiting the transport of water and metabolites to the scion.^27,28^ The effect of root restriction on shoot growth is the basis of bonsai cultivation and has been shown to have the same effect on apple scion growth as that of an dwarfing rootstock.^5^ The rate of hydraulic conductivity is lower in roots and stems of dwarfing rootstocks;^23,29^ however, this does not take into effect the smaller ‘M9’ root mass.^30^

The relative importance of the root versus the stem in rootstock-induced dwarfing has been the subject of numerous studies. Beakbane and Rogers^28^ demonstrated that scions grafted directly onto dwarfing roots were reduced in vigour and concluded that the roots alone were able to exert some effect, although the presence of a dwarfing stem enhanced this effect. Other researchers have shown that a segment of stem, or ‘interstock’, of dwarfing tissue inserted between vigorous roots and scion reduces scion vigour, with larger stem segments having a greater effect.^10,16,31,32^ Bark implants from dwarfing genotypes or even an inverted ring of bark from a vigorous genotype have been shown to dwarf the scion, providing strong evidence that the mechanism of rootstock-induced dwarfing is likely to involve the vasculature and/or bark.^33^ Hormones, soluble sugars, metabolites and nitrogen are translocated via the vascular system and have all been implicated in rootstock-induced dwarfing. Phenols affect many biological processes including auxin levels, and are concentrated in the bark of apple trees.^16^

To identify biological processes and regulatory networks that are involved with rootstock-induced dwarfing, we compared the transcriptomes of vascular-enriched tissue from dwarfing and vigorous rootstocks. Previous studies to compare apple rootstock effects on gene expression have focused on gene expression in the scion.^34,35^ While it is useful to identify scion responses to dwarfing rootstocks, the aim of our study was to identify differences in physiological states between dwarfing and vigorous rootstocks that could influence scion growth. We included the strongly dwarfing ‘M27’ in our study to identify processes that were common to two different dwarfing rootstocks.

## MATERIALS AND METHODS

### Plant material

Grafted apple (*Malus × domestica Borkh*) trees were grown in 2012–2013 for RNA collections and dry weight measurements. In a second experiment, grafted trees were grown in 2014–2015 for non-structural carbohydrate and metabolic analysis. For RNA collections and dry weight measurements, ‘Royal Gala’ (‘RG’) scions were cleft-grafted at a height of 35 cm onto 1-year-old rootstock stools of ‘M793’, ‘M9’ (clone ‘NZ9’) and ‘M27’ rootstocks (Waimea Nursery, Nelson, NZ, USA) in August (winter). In early October, scions were de-budded to a single vegetative bud and received no further pruning. Grafted trees were planted into 50 l bags containing growing medium (Growcom, Brisbane, Queensland, Australia). Irrigation was scheduled daily for 30 min (min) at dawn and dusk using an automated time controller. For the carbohydrate and metabolomics analysis, ‘RG’ scions were grafted onto either ‘M9’ or ‘RG’ rootstocks and grown as described above.

### RNA purification

Vascular-enriched tissue was collected for RNA purification in November of 2012, and January and March of 2013. These collection dates correspond to 60, 120 and 180 days after scion bud break (DABB), respectively. For each time point, four to six trees of each rootstock genotype were selected for uniform scion growth to minimise any effects because of differential tree size. Previous work has shown that the scion bud type affects both scion and rootstock growth;^3^ therefore, all RNAs were collected from compound trees with a monopodial shoot (originating from a vegetative scion bud). The outer bark was removed with a razor blade and vascular-enriched tissue was collected from the rootstock stem 2–10 cm below the graft junction and immediately snap-frozen in liquid nitrogen. Tissue was harvested between 4 and 5 h after sunrise for all time points. Total RNA was isolated as previously described.^36^ The quality and concentration of the RNA samples were assessed using a Fragment Analyzer (Advanced Analytical, Ankey, IA, USA). Only samples with a RNA integrity number value of 8 or higher were further analysed by sequencing or quantitative reverse transcriptase PCR (qRT-PCR).

### RNA sequencing, data processing and analysis

RNA from the 60 DABB (November) collection was sent to Axeq/Macrogen for library preparation and sequencing using an Illumina Hiseq 2000 (San Diego, CA, USA) instrument. RNA from six individuals of each genotype was made into separate libraries. The 18 libraries were run as a multiplexed sample on one lane to produce 100 nucleotide paired end sequence reads. The first 13 bases of all RNA sequencing (RNAseq) reads were trimmed using an in-house perl script. Adapters were removed using fastq-mcf from the ea-utils package.^37^ Quality score analysis was performed using fastqc (http://www.bioinformatics.babraham.ac.uk/projects/fastqc/) both before and after trimming. Trimmed reads with a minimum length of 30 bp and an average quality score greater than 20 were mapped to the Apple Genome V1.0-predicted coding DNA sequence (CDS) sequences (https://www.rosaceae.org/species/malus/malus_x_domestica/genome_v1.0) using bowtie2 v2.2.5 (ref. 38) using the following settings: end-to-end mapping in sensitive mode with a maximum of one mismatch per alignment. A count table was generated for each predicted CDS across all the libraries by querying for the best alignment for each sequence using samtools v1.2.^39^ Raw read counts and reads per kilobase per million values were extracted from BAM files using the multicov option of bedtools^40^ and either an in-house R script or cufflinks.^41^ The RNAseq data can be found in NCBI project PRJNA358443. Pairwise comparisons were made between ‘M9’ and ‘M793’ and ‘M27’ and ‘M793’. Differentially expressed genes (DEGs) were selected using the DEseq 2 package^42^ in BioConductor. Significant DEGs were selected using an adjusted *P* value of <0.05 and |log2 fold change|>1. Significantly over-represented gene categories in the DEGs were identified using Fisher’s exact test and visualised in Pageman using MapMan 3.5.0 and mappings for *Malus domestica.*^43^ Metabolic pathways were visualised using Kyoto Encyclopedia of Genes and Genomes (KEGG) gene ontologies.^44^ Arabidopsis orthologues were determined by BLAST against the TAIR database. Chromosome and position were determined by an in-house database. Venn diagrams were generated by Venny 1.0 (http://bioinfogp.cnb.csic.es/tools/venny/index.html).

### Gene expression by qRT-PCR

First strand complementary DNA was synthesised from 1.0 μg total RNA using oligo dT primer and Primescript Reverse Transcriptase (TaKaRa, Clontech, Mountain View, CA, USA). qRT-PCR was performed with KAPA Sybrfast qRT-PCR mastermix on a Roche 480 Light Cycler (Basel, Switzerland). For the qRT-PCR reactions, 2 μL complementary DNA (1:20 dilution) was used as a template in a reaction volume of 7 μL. For each analysis, there were four to six biological replicates of each genotype and four technical replicates of each sample. Complementary DNAs were loaded into a 384-well plate by a Biomeck liquid handling robot (Biomeck, Waltham, MA, USA) to minimise pipetting errors. PCR cycles are as follows: initial denaturation at 95 °C for 5 min, followed by 45 cycles of 94 °C for 10 s, 55 °C for 15 s, 72 °C for 10 s and a final melt curve analysis to determine whether a single product was amplified. Primers were designed by Primer 3 to span an intron (if possible) and to amplify products of 100–120 base pairs (Supplementary Table 1). For each analysis, a no complementary DNA template was included as a negative control. Actin (MDP0000752428) and MDP0000173025 were used as reference genes and gave similar results.^45^ All qRT-PCR results are shown as expression relative to actin, except for MDP0000264875. Primer efficiencies and relative expression were calculated using the Roche 480 Light Cycler software (version SW1.5).

### Dry weight measurements

After tissue was collected for RNA extractions (60, 120, 180 and 300 DABB), four to six trees of each rootstock genotype were harvested, severed at the graft junction, and then oven-dried at 60 °C to a constant mass before weighing. Dry weights of scion include scion budwood, primary axis, sylleptic shoots and leaves, while dry weights of rootstock includes roots and rootstock stem. One-way analysis of variance analysis and graphing were performed with OriginLab 8.5 (Northampton, MA, USA).

### Non-structural carbohydrate quantification

After extension growth had ceased (250 DABB), final architectural measurements were made of six ‘RG’/‘M9’ and six ‘RG’/‘RG’ grafted trees. A segment of stem tissue was collected from 20 cm above the graft junction (scion), 5 cm below the graft junction (rootstock stem) and roots. Tissue was snap-frozen in liquid nitrogen, dried in a freeze-dryer, and then ground to a fine powder. A 0.05 g subsample was extracted with 80% ethanol with Adonitol added as the internal standard and then incubated for 1 h at 60 °C. Extracted samples were centrifuged and the supernatant decanted off. The residue was re-suspended in 80% ethanol re-spun and supernatants combined. The insoluble residue was transferred into Erlenmeyer flasks and analysed for starch as per Smith *et al.*^46^ A subsample of the supernatant was taken and dried using a centrifugal concentrator; samples were then re-dissolved in ultrapure water. The sugars were analysed using DIONEX ICS-5000 Reagent-Free IC (RFIC; Thermo Fisher Scientific, Waltham, MA, USA) system with a CarboPac MA1 column with electrochemical detection.

### Secondary metabolite analysis

The ‘RG’ and ‘M9’ stem samples used for analysis by liquid chromatography–high resolution accurate mass–mass spectrometry (LC–HRAM–MS) were the same as those used for the carbohydrate analysis. There were six biological replicates and each sample was run in duplicate. The LC–HRAM–MS system was composed of a Dionex Ultimate 3000 Rapid Separation LC and a micrOTOF QII high resolution mass spectrometer (Bruker Daltonics, Bremen, Germany) fitted with an electrospray ion source. Metabolite separation by LC was achieved using a Luna Omega Polar 2.1×100 mm, 1.6 μm (Phenomenex, Auckland, New Zealand) maintained at 40 °C. The flow was 350 μL min^−1^. The solvents were A=0.2% formic acid and B=100% acetronitrile. The solvent gradient was: 10% A 90% B 0–0.5 min; linear gradient to 50% A 50% B, 0.5–12 min; linear gradient to 5% A 95% B, 12–15 min; composition held at 5% A 95% B, 15–17 min; linear gradient to 10% A, 90% B, 17–17.2 min; to return to the initial conditions before another sample injection at 20 min. The injection volume for samples and standards was 1 μL. The micrOTOF QII parameters for polyphenolic analysis were: temperature 225 °C; drying N_2_ flow 6 Lmin^−1^; nebuliser N_2_ 1.5 bar, endplate offset −500 V, mass range 100–1500 Da, acquired were acquired at 5 scans per s. Negative ion electrospray was used with a capillary voltage of +3500 V. Post-acquisition internal mass calibration used sodium formate clusters with the sodium formate delivered by a syringe pump at the start of each chromatographic analysis.

The molecular features present in each sample were found using the find-molecular-feature algorithm in the DataAnalysis (Bruker Daltonics). The find-molecular-feature process combines mass spectral signals that are related to each other (isotope clusters and molecular adducts) into single molecular features. The molecular features for each analysis were combined into a single data table using ProfileAnalysis (Bruker Daltonics). The intensity values were normalised by 'the sum of all intensities in an analysis'. To assess the distribution of metabolites in the ‘RG’ and ‘M9’ sample sets, the data table was analysed using both principal component analysis and *t*-tests using ProfileAnalysis. Metabolites that showed a |fold change|>1.2 between ‘M9’ and ‘RG’, and a *P* value<0.05, were selected for further analysis. The chemical identity of each metabolite was based on accurate mass and chemical formula calculations and authentic standards when available.

## RESULTS

### Rootstock-induced dwarfing first manifests late in first season of growth

**Figure 1:**
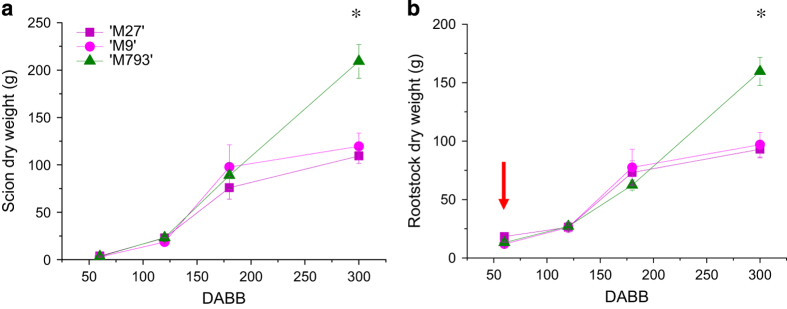
Tree dry weight accumulation during the first year of growth. ‘Royal Gala’ scions were grafted to ‘M793’ (vigorous), ‘M9’ (dwarfing) or ‘M27’ (strongly dwarfing). At each time point, six composite trees of each rootstock genotype were severed at the graft junction, (**a**) scion and (**b**) rootstock were dried and weighed. Values were compared by analysis of variance (ANOVA) and the only significant differences detected between vigorous and dwarfing rootstocks was at the final time point (*P value<0.001). Error bars are s.e. and red arrow indicates when RNA was isolated for sequencing.

Primary shoots began growth shortly after bud break and sylleptic shoots began to extend about 80 DABB. Primary and sylleptic shoot growth slowed around 200 DABB, with trees on the dwarfing rootstocks terminating earlier, consistent with earlier studies.^3^ All trees terminated extension growth by 250 DABB. No significant difference was detected in either scion or rootstock dry weight between any of the rootstock treatments until 300 DABB (Figure 1). Trees on the dwarfing rootstocks gained very little dry weight after 180 DABB, whereas trees on ‘M793’ had a constant increase in dry weight from 120 to 300 DABB.

### Genes differentially expressed between dwarfing and vigorous rootstocks

**Figure 2:**
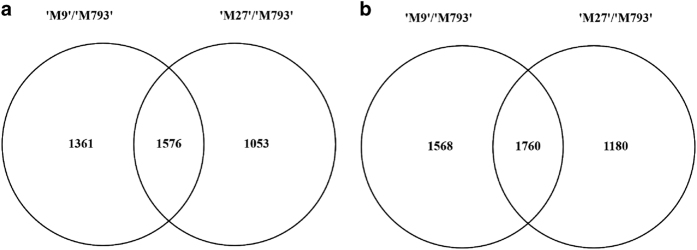
Venn diagrams showing differentially expressed genes (DEGs) between dwarfing and vigorous rootstocks. Genes with a |log2 fold change| greater than 1, between ‘M9’ (left) and ‘M27’ (right) relative to the vigorous ‘M793’, overlap show DEG common to both dwarfing genotypes. (**a**) Upregulated and (**b**) downregulated genes from vascular-enriched rootstock tissue. All rootstocks were grafted with ‘RG’ scions.

To identify differences in rootstock gene expression before any phenotypic changes to ‘RG’ scion growth were detected, we undertook a global transcriptomic analysis at 60 DABB. Vascular-enriched rootstock RNA was also extracted at time points corresponding to floral bud initiation (120 DABB) and slowing of scion growth (180 DABB). Read counts of 10 063 621 to 17 934 007 million were obtained. Using an adjusted *P* value of <0.05 as a cutoff, we identified 8880 DEGs between ‘M9’ and ‘M793’, and 7827 DEG between ‘M27’ and ‘M793’. Genes with a |log2 fold change|>1 were selected for further analysis (Figure 2). We reasoned that DEGs common to both dwarfing rootstocks could yield information about biological processes that are essential for rootstock-induced dwarfing. Compared with ‘M793’, 1576 genes were upregulated and 1760 were downregulated in both dwarfing rootstocks (Figures 2a and b).

**Figure 3:**
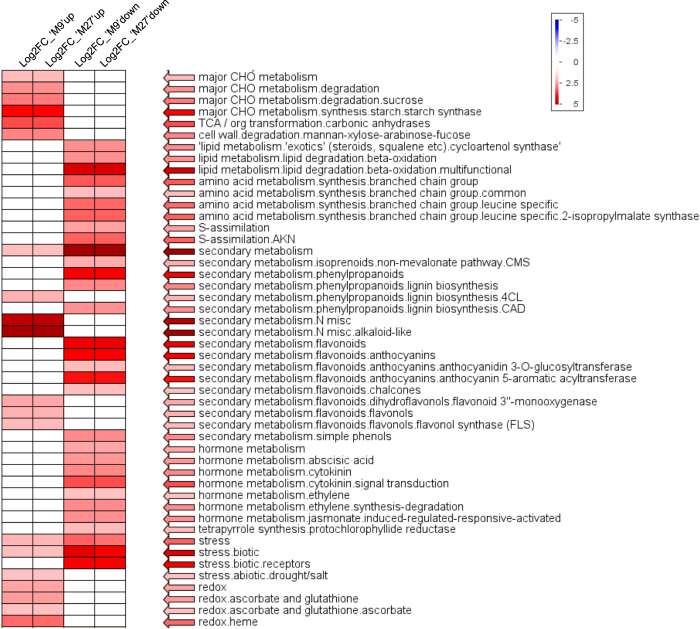
Pageman display of MapMan functional categories over-represented in genes differentially expressed between dwarfing and vigorous rootstocks. Fisher’s exact test was used to determine whether significantly more genes in a given category were over-represented (red) or under-represented (blue) in both upregulated and downregulated differentially expressed gene (DEG). Nonsignificant categories are not shown.

The 3336 DEGs common to both ‘M9’ and ‘M27’ were analysed by Fisher’s exact test using the Mapman ontology.^43^ This revealed functional categories that were over-represented in both upregulated and downregulated DEGs (Figure 3). Throughout this paper, gene expression is presented as relative to the vigorous ‘M793’. Gene ontology categories that were over-represented in upregulated DEGs were involved with carbohydrate metabolism, cell wall degradation, redox and post-translational modification. DEGs involved with amino acid and protein synthesis, secondary metabolism and hormone response were over-represented in downregulated genes. Stress response and kinase signalling genes were found in both up- and downregulated DEGs.

### Dwarfing rootstocks contain more starch and less glucose and fructose

Of the DEG involved in primary metabolism, amino acid and lipid metabolism showed a similar trend; genes promoting biosynthesis were downregulated in dwarfing rootstocks, while those promoting degradation were upregulated (Table 1). We used the KEGG and gene ontologies to visualise metabolic pathways identified as having over-represented DEGs. Genes in the KEGG pathway of fatty-acid synthesis were particularly downregulated in dwarfing rootstocks (Supplementary Figure S1).

**Figure 4:**
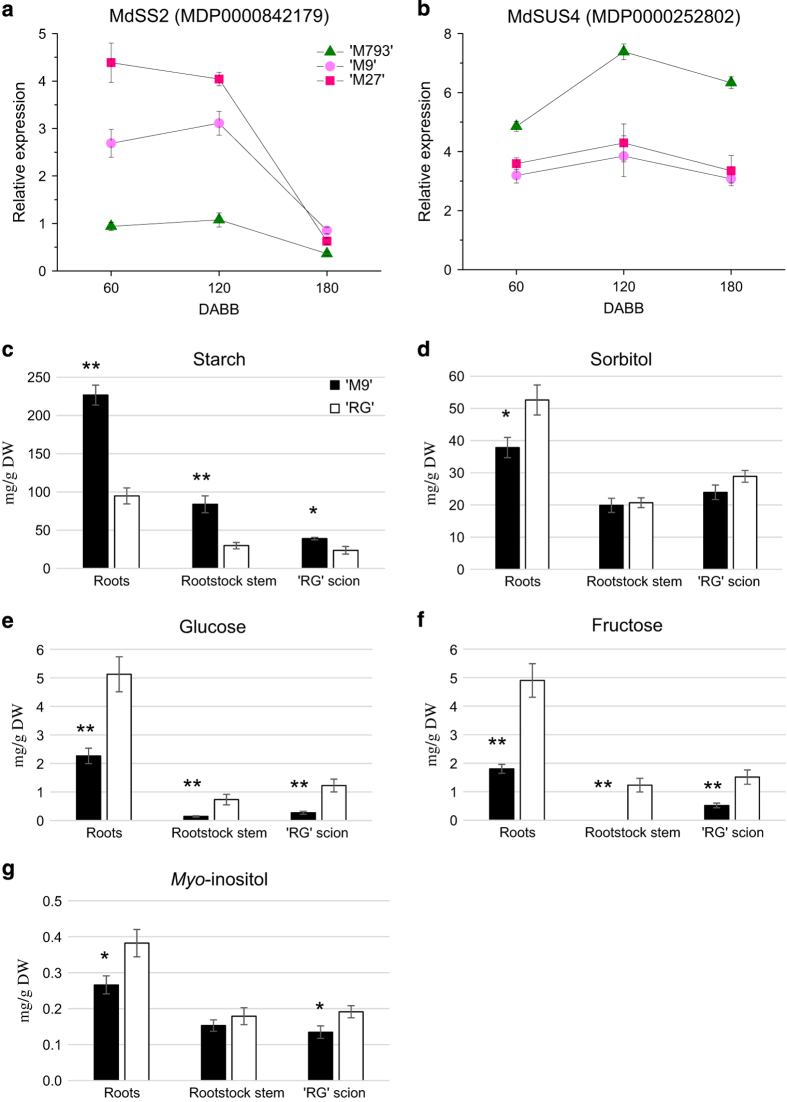
Dwarfing rootstocks accumulate more starch and less glucose and fructose. Relative expression levels of (**a**) *Mdstarch synthase 2* (*MdSS2*) and (**b**) *Mdsucrose synthase 4* (*MdSUS4*). Symbols are averages of four to six biological replicates, bars are s.e. Average values of (**c**) starch, (**d**) sorbitol, (**e**) glucose, (**f**) fructose and (**g**) *myo*-inositol in the roots, the rootstock stem and scion stem of ‘RG’/’M9’ and ‘RG’/‘RG’ grafted trees; bars indicate standard error. Values were compared by analysis of variance (ANOVA); significant differences between rootstock treatments are indicated by asterisks, *P value<0.05, **P value<0.01.

In contrast, DEG in the starch and sucrose metabolism pathway showed the opposite trend. *MdStarch synthesis* genes (*MdSS2*) were upregulated in dwarfing rootstocks, whereas starch breakdown genes *MdBeta-amylase* (*MdBAM7*) were downregulated (Table 1). Sucrose synthase (SUS) reversibly hydrolyses sucrose into fructose and UDP-glucose. *MdSUS* genes were downregulated in dwarfing genotypes (Table 1). We compared the expression of starch and sucrose metabolism genes over three time points during the year. Both *MdSS2* genes (MDP0000842179 and MDP0000283839) were expressed more highly in ‘M27’ and ‘M9’ from 60 to 120 DABB, but were at similar levels to ‘M793’ at 180 DABB, towards the end of the growing season (Figure 4a and Supplementary Figure S2). *MdSUS4* (MDP0000252802) expression was higher in ‘M793’ throughout the year, with the greatest difference at 120 DABB, the middle of summer (Figure 4b).

We next analysed non-structural carbohydrate concentrations in scion stem, rootstock stem and roots comparing ‘RG’ scions grafted to either ‘M9’ or ‘RG’ rootstocks (‘RG’/‘M9’ and ‘RG’/‘RG’, respectively). For each tissue type, the starch concentration was twice as high in ‘RG’/‘M9’ relative to ‘RG’/‘RG’ (Figure 4c). Sorbitol concentration was lower in ‘M9’ roots (Figure 4d). Glucose and fructose levels in ‘RG’/‘M9’ trees were less than half that in equivalent tissues from the homo-grafted trees (Figures 4e and f). *myo*-inositol concentrations were lower in ‘M9’ roots and ‘RG’ scion stems on ‘M9’ rootstocks (Figure 4g). Sucrose and galactose concentrations were similar in both treatments (Supplementary Figure S3). The carbohydrate analysis is consistent with the gene expression data and together indicates an imbalance of starch, glucose, fructose and *myo*-inositol in the dwarfing rootstocks relative to non-dwarfing ones. Because ‘RG’ is not normally used as a rootstock, scion growth was analysed prior to the carbohydrate analysis (250 DABB). ‘RG’/‘RG’ trees developed 60% more nodes than the ‘RG’/‘M9’ trees, largely due to homo-grafted trees having threefold more sylleptic shoots than the ‘RG’/‘M9’ (Supplementary Table S2).

### Reduced cell wall synthesis in dwarfing rootstocks

**Figure 5:**
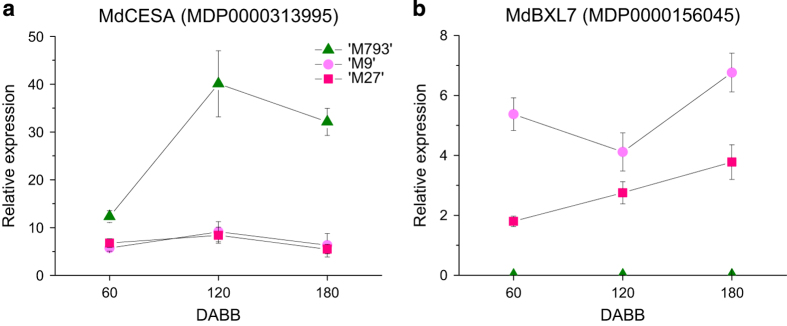
Reduced cell wall synthesis in dwarfing rootstocks. Relative expression of (**a**) *MdCellulose synthase A* (*MdCES A*), a key cell wall synthesis gene, and (**b**) *MdBeta-D-xylosidase 7* (*MdBXL7*), which promotes cell wall degradation. Symbols are averages of four to six biological replicates; bars are s.e.

Genes promoting cell wall biosynthesis and modification were downregulated in dwarfing rootstocks, whereas genes promoting cell wall hydrolysis and catabolism were upregulated. Multiple cellulose synthase genes were downregulated in dwarfing rootstocks, whereas cellulases, pectin lyase and glycosyl hydrolases were upregulated (Table 1). To investigate this further, gene expression was measured by qRT-PCR over a time course. *MdCellulose synthase A* (*MdCES A*, MDP0000313995), which encodes a key enzyme in secondary cell wall synthesis, was expressed higher in ‘M793’ throughout the year (Figure 5a). In contrast, genes promoting cell wall degradation were upregulated in dwarfing rootstocks (Table 1). *MdBeta-D-xylosidase 7* (*MdBXL7*, MDP0000156045) encodes a glycoside hydrolase involved with the degradation and reorganisation of the cell polysaccharides.^47^ *MdBXL7* was expressed much higher in the dwarfing rootstocks throughout the year (Figure 5b).

### DEG shows flux towards flavonoid and away from lignin biosynthesis in dwarfing rootstocks

**Figure 6:**
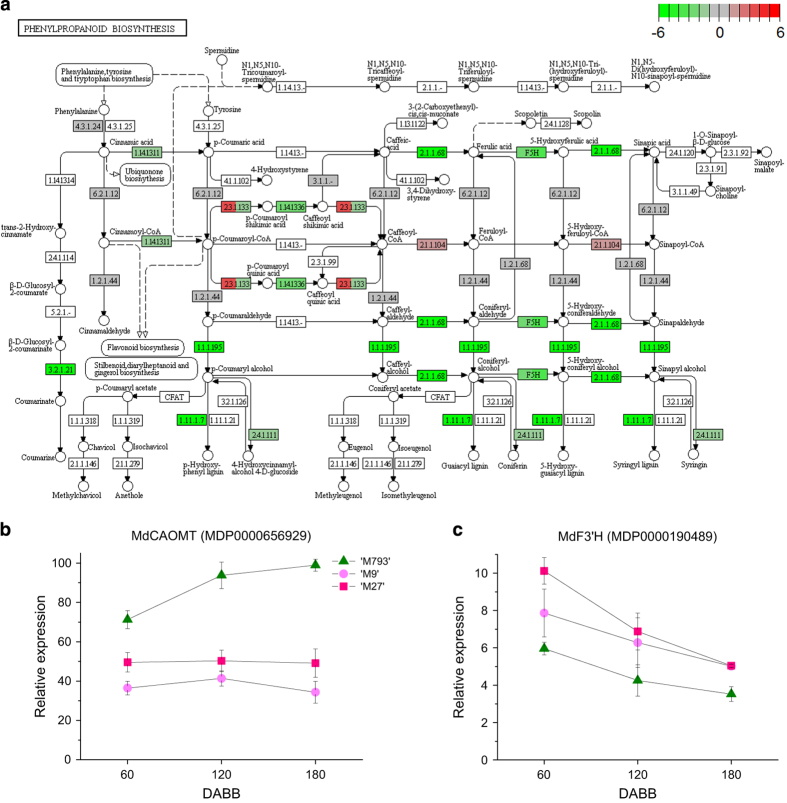
Differentially expressed gene (DEG) in phenylpropanoid biosynthesis pathways. Kyoto Encyclopedia of Genes and Genomes (KEGG) pathways showing genes that promote phenylpropanoid biosynthesis. The left side of each box represents the relative expression of ‘M27’/‘M793’, the right is ‘M9/‘M793’, red is upregulated, green is downregulated. Relative expression of (**c**) *MdCaffeic acid O-methyltransferase* (*MdCAOMT*) and (**d**) *MdFlavonoid 3’ hydroxylase* (*MdF3’H*). Symbols are averages of four to six biological replicates; bars are s.e.

Many of the secondary metabolism genes were downregulated in dwarfing rootstocks relative to ‘M793’ (Table 1). 4-coumaroyl CoA is a key branch point at which compounds divert to either the flavonoid or lignin synthesis pathway. The KEGG pathway for phenylpropanoid biosynthesis shows the upregulation of the lignin synthesis pathway in vigorous rootstock tissue (Figure 6a). *MdCaffeic acid O-methyltransferase* (*MdCAOMT*, MDP0000656929), which encodes a key enzyme in lignin biosynthesis, was expressed at much lower levels in the dwarfing rootstocks throughout the year (Figure 6b). Much of the lignin produced is incorporated into secondarily thickened cell walls, especially that of xylem cells.^48^ *MdFlavonoid 3’ hydrolases* (*MdF3’H*, MDP0000616265, MDP0000190489) were upregulated in dwarfing rootstocks (Figure 6c and Table 1).

The upregulation of *MdF3’H* in dwarfing rootstocks suggests an increase in flavonoid biosynthesis (Supplementary Figure S4). To identify any differences in secondary metabolite concentration between dwarfing and non-dwarfing rootstocks, we performed LC–HRAM–MS on stem tissue from the ‘RG’/‘M9’ and ‘RG’/‘RG’ trees used for carbohydrate analysis. The LC–HRAM–MS workflow produced a metabolite data table containing intensity values for 200 metabolites with each metabolite labelled by accurate mass *m*/*z* and liquid chromatography retention time. Forty-four metabolites that differed in concentration between ‘RG’ and ‘M9’ were selected for further analysis (Supplementary Figure S5). The majority of the compounds that could be tentatively identified were flavonoids, 19 were found in ‘M9’ and 3 in ‘RG’ (Table 2). Different isoforms of phloretin coumarylglucoside over-accumulated in both ‘M9’ and ‘RG’. There was a tenfold higher concentration of the amino-acid arginine in ‘M9’ relative to ‘RG’.

### DEG involved with hormone metabolism and signalling

Most of the DEGs involved with hormone synthesis and response were downregulated in dwarfing rootstocks (Table 1). Abscisic acid synthesis and response genes were highly downregulated in dwarfing rootstocks. Both copies of the auxin influx transporter, *LIKE AUXIN RESISTANT 2* (*MdLAX2*, MDP0000020317 and MDP0000155074) and two *Small Auxin Upregulated* (*SAUR*) genes (MDP0000737171 and MDP0000148780) were downregulated in dwarfing rootstocks. A *Grechen Hagen 3.6 gene* (*MdGH3.6,* MDP0000402444), involved with auxin homeostasis, was upregulated. *BRI-associated receptor kinase* (*MdBAK1, MDP0000218840*), part of the brassinosteroid signalling pathway, was highly upregulated in both dwarfing rootstocks, although there was no evidence of altered expression of brassinosteroid response genes. Likewise, the genes encoding ethylene signal transduction proteins were upregulated in dwarfing rootstocks, but no changes in ethylene response genes were detected. Both copies of the cytokinin signalling *histidine kinase 3* (*MdAHK3*, MDP0000181429 and MDP0000659407) were downregulated, whereas the apple homologue of *Cytokinin oxidase 7* (*MdCKX7*, MDP0000264875) was upregulated in dwarfing rootstocks. Gibberellin and jasmonic acid synthesis genes were downregulated. An oxidoreductase that promotes the synthesis of strigolactone was upregulated.

**Figure 7:**
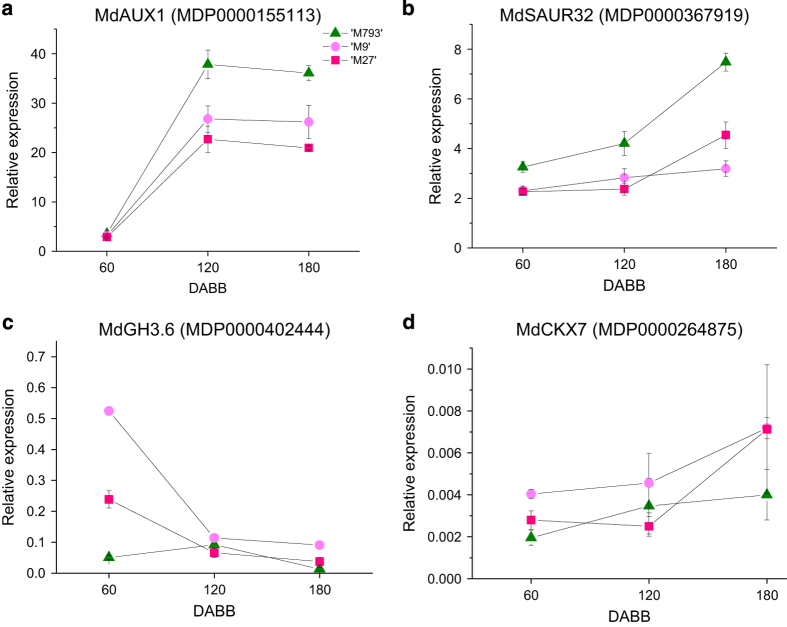
Decreased auxin and cytokinin signal transduction in dwarfing rootstocks. Relative expression of (**a**) the auxin efflux transporter *MdAUXIN RESISTANT 1* (*MdAUX1)*, auxin response genes (**b**) *MdSmall Auxin Upregulated 32 (MdSAUR32),* (**c**) *Gretchen Hagen 3.6 (GH3.6)* and (**d**) *Cytokinin dehydrogenase 7 (MdCXK7)*. Symbols are averages of four to six biological replicates; bars are s.e.

We identified other members of the MdAUX1/LAX family of auxin influx transporters and monitored expression of *MdAUXIN RESISTANT 1* (*MdAUX1,* MDP0000155113) over the year. At 60 DABB, *MdAUX1* was expressed at similar, low levels in all rootstocks. However, during the period of maximum growth from 120 to180 DABB, *MdAUX1* expression levels in ‘M793’ rose to nearly twice that of the dwarfing rootstocks’ (Figure 7a). *MdSAUR32* (MDP0000367919) expression was reduced in dwarfing rootstocks throughout the year (Figure 7b). *MdGH3.6* expression was higher in the dwarfing rootstocks, but only at 60 DABB (Figure 7c). *MdCKX7* was upregulated in both ‘M9’ and ‘M27’ (Figure 7d).

### Redox status, signalling kinases and stress response

Most of the DEG genes regulating cellular redox homeostasis were upregulated in dwarfing rootstocks. Both copies of *Dehydroascorbate reductase*, central to ascorbate and glutathione metabolism, were expressed at much higher levels in dwarfing rootstocks (Table 1). Three peroxidase genes were highly upregulated in dwarfing rootstocks, indicating response to oxidative stress. The only redox genes that were downregulated were three encoding 2OG-Fe (II) oxygenase proteins.

Genes encoding leucine-rich repeat transmembrane kinases were upregulated in both ‘M793’ and in the dwarfing rootstocks (Supplementary Table S3). Type VIII leucine-rich repeats were upregulated in dwarfing rootstocks, whereas type XII were predominantly upregulated in ‘M793’. The only functional category that was upregulated only in ‘M9’ and ‘M27’ were the Wall-associated kinases (WAK), which bind pectin and are often associated with response to biotic stress. All five of the *WAK* genes were expressed at significantly higher levels in the dwarfing rootstocks. This result was confirmed by qPCR (Supplementary Figure S6). Biotic stress response genes were upregulated in both ‘M793’ and the dwarfing rootstocks.

## DISCUSSION

### Reduced biogenesis in dwarfing rootstocks

The results of this study indicate that apple dwarfing rootstocks are in a general state of reduced cell growth and metabolism long before any visible effects are apparent in either rootstock or scion. Amino acid, lipid and cell wall biosynthesis pathways are downregulated in dwarfing rootstocks, whereas the degradation pathways of these compounds are upregulated. Lignin and cellulose biosynthesis pathways are highly downregulated in dwarfing rootstocks, consistent with reduced cell wall synthesis and modification, and possibly a contributing factor to the reduced root mass and hydraulic conductance observed in dwarfing rootstocks.

### Imbalanced carbon allocation influences growth and development

Normally, starch reserves in roots are catabolized when carbon for metabolic pathways or glycolysis is limiting. We found that ‘M9’ accumulated large amounts of starch in roots and stem, yet had very low levels of the glucose and fructose relative to ‘RG’. Even ‘RG’/‘M9’ scions accumulated more starch and less glucose and fructose than scions in the homo-grafted trees, indicating a non-autonomous effect of ‘M9’ on carbohydrate allocation. *Myo*-inositol concentrations were also significantly lower in ‘M9’roots and scions on ‘M9’ rootstocks. *Myo*-inositol is commonly used in tissue culture media and has been shown to promote apple and pear root and shoot growth in a dosage-sensitive manner.^49^ A number of reviews have illustrated the role of sugars not just as nutrients, but also as signalling molecules capable of sensing nutrient status and coordinating growth and development accordingly.^50–54^ For example, sugar promotes lateral meristem outgrowth in rose,^55^ pea^56^ and sorghum.^57^ The low concentration of glucose, fructose and *myo*-inositol in trees with ‘M9’ rootstocks would have a significant impact on the physiology of both rootstock and scion.

Colby^58^ used double-grafted apple trees to demonstrate that interstocks (stem segments) of ‘M9’ grafted onto seedling roots retained high concentrations of starch in the roots and reduced growth of scion, whereas interstocks of an invigorating genotype depleted all starch from ‘M9’ roots and led to vigorous growth of the scion. Higher starch concentrations in citrus roots have also been correlated with reduced scion growth.^59^ On the basis of these observations and our findings, we propose that apple dwarfing rootstocks are impaired in sensing and/or maintaining the balance between starch reserves, cellulose and hexose sugars for glycolysis and cell metabolism. Because of this defect, dwarfing rootstocks act as ‘super sinks’, holding excess starch reserves at the expense of both root and scion growth.

Competition between sink tissues is a well-documented phenomenon in fruiting trees. Guitton *et al.*^60^ described molecular signatures of carbon starvation and oxidative stress in apple buds from heavily flowering trees with a high propensity for biennial bearing. They suggest that competition for carbohydrates between developing fruit (strong sinks) and nearby apical buds (weaker sinks) leads to a local carbon depletion and reduced cellular activity in the vegetative meristems, thus blocking the onset of floral development.

### New insight into reduced auxin transport in dwarfing rootstocks

Researchers have long speculated that rootstock-induced dwarfing involved reduced auxin transport.^1^ Reduced auxin transport in ‘M9’ was finally proven late last century,^17,18^ but few advances have been made since towards identifying the underlying mechanism responsible. The results of our RNAseq analysis indicated that the phenylpropanoid pathway showed flux towards flavonoid biosynthesis and away from lignin biosynthesis in dwarfing rootstocks. LC–HRAM-–MS data confirmed the over-accumulation of flavonoids in ‘M9’ relative to ‘RG’. Both genetic and metabolomic analyses have provided strong evidence that flavonoids inhibit polar auxin transport.^61–68^ Plants that over-accumulate flavonoids have reduced auxin transport and dwarfed phenotypes,^67,68^ and those that are blocked in flavonoid synthesis or glycosylation (CHS) have increased auxin transport and also show abnormal root and shoot phenotypes.^62–66^ Chalcone synthase catalyses the first committed step in flavonoid biosynthesis. Silencing of the apple *CHS *gene completely removed many of the same flavonoid compounds that over-accumulated in ‘M9’.^66^ Future research is needed to explore the role of flavonoids in rootstock-induced dwarfing. The reduced expression of *MdAUX1* and *MdLAX2* observed in the dwarfing rootstocks may also contribute to reduced polar auxin transport.

### Interactions between sugar and hormone signalling

Auxin and cytokinin act antagonistically to regulate root and shoot growth, the outgrowth of axillary meristems and the synthesis and transport of one another.^69^ Both have been implicated in the mode of rootstock-induced dwarfing.^16^ Downregulation of *SAUR* genes in dwarfing rootstocks indicates a reduced auxin response. Expression of *MdAUX1* and the auxin conjugating *MdGH3.6* was variable over the season, which likely reflects the fact that auxin signalling is tightly regulated by feedback, feedforward and cross-talk with other signalling pathways. Cytokinin signal transduction genes were downregulated, which is consistent with previous reports of lower cytokinin concentrations in ‘M9’ and ‘M27’ xylem sap.^70^ *MdCKX7*, which encodes a cytokinin-degrading enzyme, was upregulated in dwarfing rootstocks. Overexpression of *CKX* genes in *Arabidopsis* and tobacco results in slow-growing, dwarfed shoots and a reduction in soluble sugars.^71–73^ Transgenic manipulation of cytokinin activity has implicated cytokinin in regulating sink strength in storage organs.^73–75^

The xylem sap of ‘M9’ and ‘M27’ has higher ABA concentrations than that of vigorous rootstocks.^22^ In contrast, we found that multiple ABA biosynthesis and response genes were downregulated in dwarfing rootstocks. Glucose increases expression of the ABA biosynthetic genes (*ABA1-ABA3*), which were also identified as *glucose insensitive* (*gin*) mutants.^76–78^ Numerous studies have demonstrated a clear connection between sugar-sensing pathways and hormone metabolism and signalling.^50,79–82^ Sugar promotes auxin biosynthesis^83–85^ and polar auxin transport.^86,87^ Glucose and auxin transcriptionally regulate many of the same genes and appear to act synergistically in plant development.^88^ Cytokinin and glucose also share many transcriptional targets.^89^ The *gin2* mutant, which is defective in glucose sensing, is hypersensitive to cytokinin and insensitive to auxin.^75^ It is unclear whether the reduced glucose concentrations in dwarfing rootstocks are the cause or result of altered hormone levels or signalling, but changes to either would have a profound impact on growth and development of roots and shoots.

A recent publication comparing the transcriptomes of genetically identical *Malus* roots that had been grafted to different scion genotypes also found that hormone signal transduction and sugar metabolism genes were highly represented in the DEGs.^90^ The authors concluded that the scion genotype can affect root phenotype by altering sugar metabolism, and auxin and cytokinin signalling. Our results indicate that the rootstock genotype influences scion growth by the same signalling pathways.

### Genetic variation between ‘M9’ and ‘M27’ may influence gene expression

The aim of this study was to identify DEGs common to two different dwarfing rootstocks. However, it is worth noting that there were a large number of DEGs that were unique to ‘M9’ or ‘M27’, and even DEGs that were common to both did not always show the same degree of differential expression. ‘M9’ is a parent of ‘M27’,^11^ and while both contain the dwarfing loci *Dw1* and *Dw2*, the non-dwarfing alleles of these loci, as well as many other unlinked loci differ between these two genotypes.^12–15^ Harrison *et al.*^15^ have reported that the specific allelic combinations at *Dw1*, *Dw2* and a region of LG13 influence the expression of rootstock-induced dwarfing. Allelic variation at these or other loci could be responsible for the differences in gene expression observed between ‘M9’ and ‘M27’. We could not identify any obvious candidate genes that would affect carbohydrate metabolism or flavonoid biosynthesis within either the *Dw1* or *Dw2* mapping intervals. Further research is needed to identify the genetic basis for the transcriptional changes identified in this study.

## CONCLUSIONS

The most consistent manifestation of apple dwarfing rootstocks is earlier termination of primary axis and sylleptic shoot growth and reduced biomass accumulation in both roots and shoots. This generally manifests within the first year of growth after grafting and becomes more pronounced with successive growth seasons and fruiting. Our results indicate that dwarfing rootstocks do not respond to sugar depletion and eventually run out of carbon to support growth in both roots and scion. By holding excess starch reserves, apple dwarfing rootstocks would be unable to provide carbon to the scion in early spring, before the scion becomes a source of fixed carbon. Once the tree began fruiting, the developing fruit would create strong carbon sinks, further increasing competition for carbon by vegetative meristems. On the basis of our findings, we propose that excess flavonoids and reduced *MdAUX1* and *MdLAX2* expression contribute to the reduced auxin transport observed in dwarfing rootstocks.

## Figures and Tables

**Table 1:** Selection of DEG common to both 'M9' and 'M27' relative to 'M793'


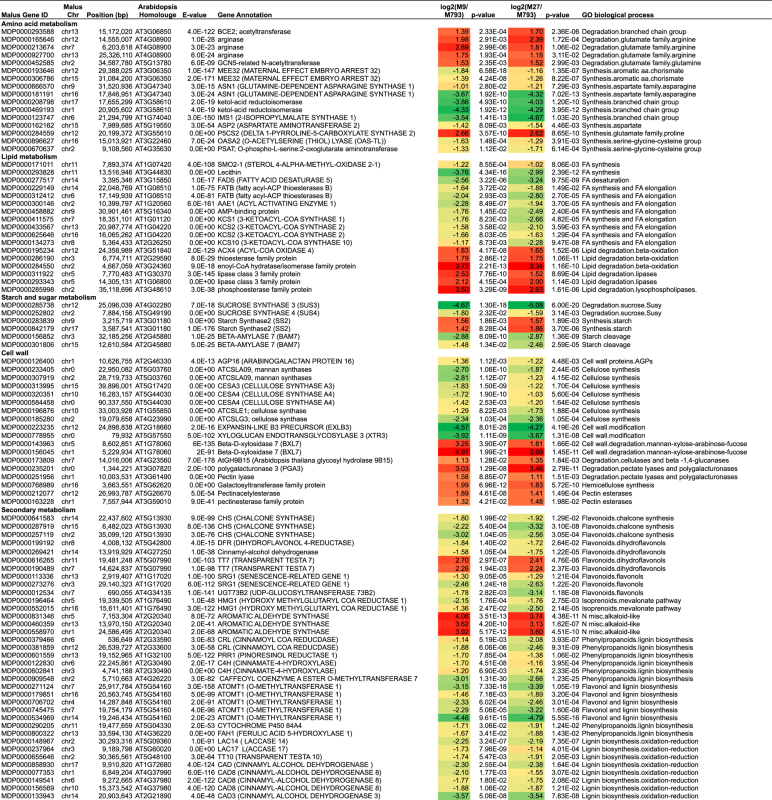
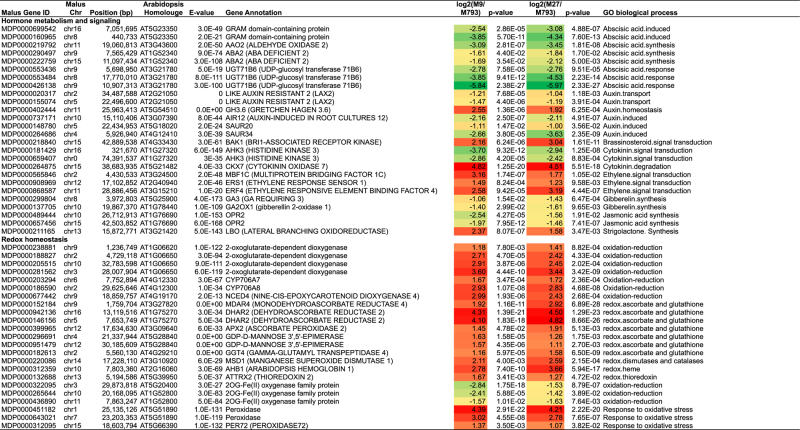

Abbreviations: Chr, chromosome; CHS, chalcone synthase; DEG, differentially expressed gene; GO, gene ontology; M9*, *Malling 9; RG, Royal Gala.

Data are log base 2 relative to ‘M793’ and is based on six biological replicates per genotype. Chromosome 0 indicates an unmapped gene model. Red/orange indicates upregulation in dwarfing rootstocks; green indicates downregulation.

**Table 2:** Results of LC–HRAM–MS analysis of ‘RG’ and ‘M9’ stem tissue from grafted trees

*Annotation*	*Type*	*Metabolite*	*'RG'*	*'M9'*	*FC 'M9'/'RG'*
Arginine	Amino acid	0.58 min: 521.326 *m*/*z*	35.0	355.5	10.16
Eriodictyol-hexoside	Flavonoid	5.35 min: 449.109 *m*/*z*	142.2	952.6	6.70
Eriodictoyl	Flavonoid	5.35 min: 287.059 *m*/*z*	74.6	470.7	6.31
Phloretin coumarylglucoside (?)	Flavonoid	11.09 min: 581.162 *m*/*z*	118.9	514.1	4.33
Isorhamnetin ?-rhamnoside	Flavonoid	7.04 min: 461.109 *m*/*z*	167.7	490.0	2.92
Procyanidin tetramer	Flavonoid	4.41 min: 1153.258 *m*/*z*	31.3	71.7	2.29
Quercetin ?-rhamnoside	Flavonoid	6.90 min: 447.093 *m*/*z*	77.9	173.9	2.23
p-coumaryl quinic acid	Phenolic acid	4.01 min: 337.095 *m*/*z*	616.4	1362.9	2.21
Frag-p-coumaryl quinic acid	Phenolic acid	4.01 min: 173.048 *m*/*z*	145.2	302.3	2.08
Phloretin-2'-O-xyloglucoside	Flavonoid	6.10 min: 567.170 *m*/*z*	368.5	726.3	1.97
Kaempferol-?-pentoside	Flavonoid	6.97 min: 417.084 *m*/*z*	129.6	254.6	1.96
Procyanidin B2	Flavonoid	3.42 min: 577.133 *m*/*z*	262.6	450.5	1.72
Procyanidin dimer (B5?)	Flavonoid	5.37 min: 577.132 *m*/*z*	69.8	111.3	1.60
Kaempferol-?-pentoside	Flavonoid	6.71 min: 417.084 *m*/*z*	84.4	134.5	1.59
Procyanidin trimer	Flavonoid	4.17 min: 865.188 *m*/*z*	224.5	347.4	1.55
Epicatechin	Flavonoid	3.83 min: 289.074 *m*/*z*	1281.6	1948.1	1.52
Kaempferol 3-rhamnoside	Flavonoid	7.17 min: 431.098 *m*/*z*	355.3	506.2	1.42
Mearnsetin-glycoside	Flavonoid	6.29 min: 477.103 *m*/*z*	2155.5	2949.5	1.37
Quercetin 3-arabinoside adduct	Flavonoid	6.18 min: 867.152 *m*/*z*	119.8	158.5	1.32
Quercetin 3-xyloside	Flavonoid	5.89 min: 433.078 *m*/*z*	942.4	1189.5	1.26
Quercetin 3-rhamnoside	Flavonoid	6.35 min: 447.093 *m*/*z*	3542.5	4338.7	1.22
Quercetin 3-arabinoside	Flavonoid	6.23 min: 433.078 *m*/*z*	1554.4	1867.8	1.20
Phloretin ?-pentoside	Flavonoid	7.53 min: 405.120 *m*/*z*	95.0	109.5	1.15
Myricetin 3-pentoside	Flavonoid	4.65 min: 449.073 *m*/*z*	135.2	80.4	−1.68
Sucrose(quinic) adduct		0.67 min: 533.171 *m*/*z*	313.0	175.1	−1.79
Quercetin 3-galactoside	Flavonoid	5.54 min: 463.088 *m*/*z*	389.3	202.7	−1.92
Quinic acid	Organic acid	0.67 min: 191.059 *m*/*z*	1069.8	447.0	−2.39
Oxo-dihydroxy-urs-12-ene-28-oic acid	Terpene	14.92 min: 485.326 *m*/*z*	3465.1	495.2	−7.00
Trihydroxy-urs-12-ene-28-oic acid	Terpene	14.05 min: 487.341 *m*/*z*	689.1	94.4	−7.30
Phloretin coumarylglucoside (?)	Flavonoid	9.74 min: 581.166 *m*/*z*	640.8	38.1	−16.83

Abbreviations: LC–HRAM–MS, liquid chromatography–high-resolution accurate mass–mass spectrometry; M9*, *Malling 9; RG, Royal Gala.

Values are the means of two technical replicates of each individual and six biological replicates each of ‘RG’ and ‘M9’. The liquid chromatography retention time and accurate mass (*m*/*z*) for each metabolite is given. Concentrations are in units per mg dry weight, FC indicates fold change of 'M9' relative to 'RG'. The annotation is based on accurate mass and chemical formula calculations and authentic standards when available. The question marks indicate that a clear annotation could not be made.

## References

[bib1] Beakbane AB, Thompson EC. Anatomical studies of stems and roots of hardy fruit trees II. The internal structure of the roots of some vigourous and some dwarfing apple rootstocks, and the correlation of strucutre with vigour. J Pomol Hortic Sci 1939; 17: 141–149.

[bib2] van Hooijdonk B, Woolley D, Warrington I, Tustin S. Rootstocks modify scion architecture, endogenous hormones, and root growth of newly grafted 'royal gala' apple trees. J Am Soc Hortic Sci 2011; 136: 93–102.

[bib3] Foster TM, Hooijdonk BMV, Friend AP, Seleznyova AN, Andrew RGM. Apple rootstock-induced dwarfing is strongly influenced by growing environment. J Hortic 2016; 3: 180–188.

[bib4] Costes E, Salles J, Garcia G. Growth and branching patterns along the main axis of two apple cultivars grafted on two different rootstocks. Acta Hort 2001; 557: 131–139.

[bib5] van Hooijdonk BM, Woolley D, Warrington I, Tustin S. Architectural development of ‘Royal Gala’ apple scions in response to rootstock, root restriction, and benzylaminopurine. Acta Hort 2006; 727: 561–568.

[bib6] van Hooijdonk BM, Woolley DJ, Warrington IJ, Tustin DS. Initial alteration of scion architecture by dwarfing apple rootstocks may involve shoot-root-shoot signalling by auxin, gibberellin, and cytokinin. J Hortic Sci Biotechnol 2010; 85: 59–65.

[bib7] Seleznyova AN, Thorp TG, White M, Tustin S, Costes E. Application of architectural analysis and AMAPmod methodology to study dwarfing phenomenon: the branch structure of 'Royal Gala' apple grafted on dwarfing and non-dwarfing rootstock/interstock combinations. Ann Bot 2003; 91: 665–672. 12714365 10.1093/aob/mcg072 PMC4242355

[bib8] Foster TM, Watson AE, van Hooijdonk BM, Schaffer RJ. Key flowering genes including FT-like genes are upregulated in the vasculature of apple dwarfing rootstocks. Tree Genet Genomes 2014; 10: 189–202.

[bib9] Seleznyova AN, Tustin S, White M, Costes E. Analysis of the earliest-observed expression of dwarfing rootstock effects on young apple trees, using application of Markovian models. Acta Hort 2007; 732: 79–84.

[bib10] Seleznyova AN, Tustin DS, Thorp TG. Apple dwarfing rootstocks and interstocks affect the type of growth units produced during the annual growth cycle: Precocious transition to flowering affects the composition and vigour of annualshoots. Ann Bot 2008; 101: 679–687. 18263898 10.1093/aob/mcn007 PMC2710180

[bib11] Hatton RG. 'Paradise' apple stocks. J R Hortic Soc 1917; 42: 361–399.

[bib12] Pilcher RLR, Celton J-M, Gardiner SE, Tustin DS. Genetic markers linked to the dwarfing trait of apple rootstock ‘Malling 9’. J Am Soc Hortic Sci 2008; 133: 100–106.

[bib13] Fazio G, Wan Y, Kviklys D et al. Dw2, a new dwarfing locus in apple rootstocks and its relationship to induction of early bearing in apple scions. J Am Soc Hortic Sci 2014; 139: 87–98.

[bib14] Foster TM, Celton J-M, Chagné D, Tustin DS, Gardiner SE. Two quantitative trait loci, Dw1 and Dw2, are primarily responsible for rootstock-induced dwarfing in apple. Hortic Res 2015; 2: 15001. 26504562 10.1038/hortres.2015.1 PMC4595989

[bib15] Harrison N, Harrison RJ, Barber-Perez N et al. A new three-locus model for rootstock-induced dwarfing in apple revealed by genetic mapping of root bark percentage. J Exp Bot 2016; 67: 1871–1881. 26826217 10.1093/jxb/erw001 PMC4783367

[bib16] Lockard RG, Schneider GW. Stock and scion relationships and the dwarfing mechanism in apple. Hort Rev 1981; 3: 315–375.

[bib17] Soumelidou KMD, Battey NH, Barnett JR, John P. Auxin transport capacity in relation to the dwarfing effect of apple rootstocks. J Hortic Sci 1994; 69: 719–725.

[bib18] Kamboj JS, Browning G, Quinlan JD, Blake PS, Baker DA. Polar transport of [3H]-IAA in apical shoot segments of different apple rootstocks. J Hortic Sci 1997; 72: 773–780.

[bib19] Michalczuk L. Indole-3-acetic acid level in wood, bark and cambial sap of apple rootstocks differing in growth vigour. Acta Physiol Plant 2002; 24: 131–136.

[bib20] van Hooijdonk BM, Woolley DJ, Warrington IJ, Tustin DS. Initial alteration of scion architecture by dwarfing apple rootstocks may involve shoot-root-shoot signalling by auxin, gibberellin, and cytokinin. J Hortic Sci Biotechnol 2010; 85: 59–65.

[bib21] Richards D, Thompson WK, Pharis RP. The influence of dwarfing interstocks on the distribution and metabolism of xylem-applied [3H]Gibberellin A4 in apple. Plant Physiol 1986; 82: 1090–1095. 16665139 10.1104/pp.82.4.1090 PMC1056263

[bib22] Kamboj JS, Browning G, Blake PS, Quinlan JD, Baker DA. GC-MS-SIM analysis of abscisic acid and indole-3-acetic acid in shoot bark of apple rootstocks. Plant Growth Regul 1999; 28: 21–27.

[bib23] Tworkoski T, Fazio G. Effects of size-controlling apple rootstocks on growth, abscisic acid, and hydraulic conductivity of scion of different vigor. Int J Fruit Sci 2015; 15: 369–381.

[bib24] Beakbane AB. Anatomical structure in relation to rootstock behaviour. Proc 13th Int Hort Congr 1952, 152–157.

[bib25] Soumelidou K, Battey NH, John P, Barnett JR. The anatomy of the developing bud union and its relationship to dwarfing in apple. Ann Bot 1994; 74: 605–611.

[bib26] Simons RK, Chu MC. Graft union characteristics of 'M.26' apple rootstock combined with ‘Red Delicious’ strains—Morphological and anatomical development. Sci Hortic 1985; 25: 49–59.

[bib27] Knight RC. Water relations of apples. Anual Report for the East Malling Research Station for 1925. East Malling Research Station: East Malling, UK, 1926, 55–26.

[bib28] Beakbane AB, Rogers WS. The relative importance of stem and root in determining rootstock influence in apples. J Hortic Sci 1956; 31: 99–110.

[bib29] Atkinson CJ, Else MA, Taylor L, Dover CJ. Root and stem hydraulic conductivity as determinants of growth potential in grafted trees of apple (*Malus pumila* Mill.). J Exp Bot 2003; 54: 1221–1229. 12654873 10.1093/jxb/erg132

[bib30] Webster AD. Vigour mechanisms in dwarfing rootstocks for temperate fruit trees. Acta Hort 2004; 658: 29–40.

[bib31] Tukey H, Brase K. The dwarfing effect of an intermediate stem-piece of malling IX apple. Proc Am Soc Hortic Sci 1944; 42: 357–364.

[bib32] Parry MS, Rogers WS. Effects of interstock length and vigour on the field performance of Cox’s Orange Pippin apples. J Hortic Sci 1972; 47: 97–105.

[bib33] Roberts R. Ring grafting and stock effect. Proc Am Soc Hortic Sci 1934; 32: 328–329.

[bib34] Jensen P, Makalowska I, Altman N et al. Rootstock-regulated gene expression patterns in apple tree scions. Tree Genet Genomes 2010; 6: 57–72.

[bib35] Jensen P, Rytter J, Detwiler E, Travis J, McNellis T. Rootstock effects on gene expression patterns in apple tree scions. Plant Mol Biol 2003; 53: 493–511. 15010615 10.1023/B:PLAN.0000019122.90956.3b

[bib36] Janssen B, Thodey K, Schaffer R et al. Global gene expression analysis of apple fruit development from the floral bud to ripe fruit. BMC Plant Biol 2008; 8: 16. 18279528 10.1186/1471-2229-8-16 PMC2287172

[bib37] Aronesty E. Command-line tools for processing biological sequencing data, 2011. https://expressionanalysis.github.io/ea-utils/.

[bib38] Langmead B, Salzberg SL. Fast gapped-read alignment with Bowtie 2. Nat Methods 2012; 9: 357–359. 22388286 10.1038/nmeth.1923 PMC3322381

[bib39] Li H, Handsaker B, Wysoker A et al. The Sequence Alignment/Map format and SAMtools. Bioinformatics 2009; 25: 2078–2079. 19505943 10.1093/bioinformatics/btp352 PMC2723002

[bib40] Quinlan AR, Hall IM. BEDTools: a flexible suite of utilities for comparing genomic features. Bioinformatics 2010; 26: 841–842. 20110278 10.1093/bioinformatics/btq033 PMC2832824

[bib41] Trapnell C, Williams BA, Pertea G et al. Transcrpt assembly and quantification by RNA-Seq reveals unannotated transcripts and isoform switching during cell differentiation. Nat Biotechnol 2010; 28: 511–515. 20436464 10.1038/nbt.1621 PMC3146043

[bib42] Smyth GK. Limma: linear models for microarray data. In: Gentleman R, Carey V SD, Irizarry R, Huber W (eds). Bioinformatics and Computational Biology Solutions using R and Bioconductor. Springer: New York, NY, USA, 2005, pp 397–420.

[bib43] Thimm O, Bläsing O, Gibon Y et al. mapman: a user-driven tool to display genomics data sets onto diagrams of metabolic pathways and other biological processes. Plant J 2004; 37: 914–939. 14996223 10.1111/j.1365-313x.2004.02016.x

[bib44] Kanehisa M, Araki M, Goto S et al. KEGG for linking genomes to life and the environment. Nucleic Acids Res 2008; 36 (suppl 1): D480–D484. 18077471 10.1093/nar/gkm882 PMC2238879

[bib45] Bowen J, Ireland HS, Crowhurst R et al. Selection of low-variance expressed Malus x domestica (apple) genes for use as quantitative PCR reference genes (housekeepers). Tree Genet Genomes 2014; 10: 751–759.

[bib46] Smith GS, Clark CJ, Boldingh HL. Seasonal accumulation of starch by components of the kiwifruit vine. Ann Bot 1992; 70: 19–25.

[bib47] Minic Z. Physiological roles of plant glycoside hydrolases. Planta 2007; 227: 723–740. 18046575 10.1007/s00425-007-0668-y

[bib48] Douglas CJ. Phenylpropanoid metabolism and lignin biosynthesis: from weeds to trees. Trends Plant Sci 1996; 1: 171–178.

[bib49] Toma RS, Danial GH, Habash ANY. *In vitro* morphogenetic response of apple (*Malus domestica* Borkh.) and pear (*Pyrus cummunis* L.) to the elevated levels of copper and myo-inositol. Acta Agrobot 2012; 65: 43–48.

[bib50] Eveland AL, Jackson DP. Sugars, signalling, and plant development. J Exp Bot 2012; 63: 3367–3377. 22140246 10.1093/jxb/err379

[bib51] Bolouri Moghaddam MR, Van den Ende W. Sugars, the clock and transition to flowering. Front Plant Sci 2013; 4. 10.3389/fpls.2013.00022 PMC3572515 23420760

[bib52] Dobrenel T, Marchive C, Azzopardi M et al. Sugar metabolism and the plant target of rapamycin kinase: a sweet operaTOR? Front Plant Sci 2013; 4: 93. 23641244 10.3389/fpls.2013.00093 PMC3640205

[bib53] Lastdrager J, Hanson J, Smeekens S. Sugar signals and the control of plant growth and development. J Exp Bot 2014; 65: 799–807. 24453229 10.1093/jxb/ert474

[bib54] Rolland F, Baena-Gonzalez E, Sheen J. Sugar sensing and signaling in plants: conserved and novel mechanisms. Annu Rev Plant Biol 2006; 57: 675–709. 16669778 10.1146/annurev.arplant.57.032905.105441

[bib55] Rabot A, Henry C, Ben Baaziz K et al. Insight into the role of sugars in bud burst under light in the rose. Plant Cell Physiol 2012; 53: 1068–1082. 22505690 10.1093/pcp/pcs051

[bib56] Mason MG, Ross JJ, Babst BA, Wienclaw BN, Beveridge CA. Sugar demand, not auxin, is the initial regulator of apical dominance. Proc Natl Acad Sci USA 2014; 111: 6092–6097. 24711430 10.1073/pnas.1322045111 PMC4000805

[bib57] Kebrom TH, Mullet JE. Photosynthetic leaf area modulates tiller bud outgrowth in sorghum. Plant Cell Environ 2015; 38: 1471–1478. 25496467 10.1111/pce.12500

[bib58] Colby HL. Stock-scion chemistry and the fruiting relationships in apple trees. Plant Physiol 1935; 10: 483–498. 16653289 10.1104/pp.10.3.483 PMC439143

[bib59] Mendel K, Cohen A. Starch level in the trunk as a measure of compatibility between stock and scion in citrus. J Hortic Sci 1967; 42: 231–241.

[bib60] Guitton B, Kelner JJ, Celton JM et al. Analysis of transcripts differentially expressed between fruited and deflowered ‘Gala’ adult trees: a contribution to biennial bearing understanding in apple. BMC Plant Biol 2016; 16: 1–22. 26924309 10.1186/s12870-016-0739-y PMC4770685

[bib61] Peer WA, Murphy AS. Flavonoids and auxin transport: modulators or regulators? Trends Plant Sci 12: 556–563. 18198522 10.1016/j.tplants.2007.10.003

[bib62] Murphy A, Peer WA, Taiz L. Regulation of auxin transport by aminopeptidases and endogenous flavonoids. Planta 2000; 211: 315–324. 10987549 10.1007/s004250000300

[bib63] Brown DE, Rashotte AM, Murphy AS et al. Flavonoids act as negative regulators of auxin transport *in vivo *in Arabidopsis. Plant Physiol 2001; 126: 524–535. 11402184 10.1104/pp.126.2.524 PMC111146

[bib64] Kuhn BM, Geisler M, Bigler L, Ringli C. Flavonols accumulate asymmetrically and affect auxin transport in *Arabidopsis*. Plant Physiol 2011; 156: 585–595. 21502189 10.1104/pp.111.175976 PMC3177260

[bib65] Buer CS, Kordbacheh F, Truong TT, Hocart CH, Djordjevic MA. Alteration of flavonoid accumulation patterns in transparent testa mutants disturbs auxin transport, gravity responses, and imparts long-term effects on root and shoot architecture. Planta 2013; 238: 171–189. 23624937 10.1007/s00425-013-1883-3

[bib66] Dare AP, Tomes S, Jones M et al. Phenotypic changes associated with RNA interference silencing of chalcone synthase in apple (*Malus × domestica*). Plant J 2013; 74: 398–410. 23398045 10.1111/tpj.12140

[bib67] Yin R, Han K, Heller W et al. Kaempferol 3-O-rhamnoside-7-O-rhamnoside is an endogenous flavonol inhibitor of polar auxin transport in *Arabidopsis* shoots. New Phytol 2014; 201: 466–475. 24251900 10.1111/nph.12558 PMC4260840

[bib68] Besseau S, Hoffmann L, Geoffroy P, Lapierre C, Pollet B, Legrand M. Flavonoid accumulation in *Arabidopsis *repressed in lignin synthesis affects auxin transport and plant growth. Plant Cell 2007; 19: 148–162. 17237352 10.1105/tpc.106.044495 PMC1820963

[bib69] Müller D, Leyser O. Auxin, cytokinin and the control of shoot branching. Ann Bot 2011; 107: 1203–1212. 21504914 10.1093/aob/mcr069 PMC3091808

[bib70] Kamboj JS, Browning G, Blake PS, Quinlan JD, Baker DA. Identification and quantitation by GC-MS of zeatin and zeatin riboside in xylem sap from rootstock and scion of grafted apple trees. Plant Growth Regul 1999; 28: 199–205.

[bib71] Werner T, Motyka V, Laucou V, Smets R, Van Onckelen H, Schmülling T. Cytokinin-deficient transgenic arabidopsis plants show multiple developmental alterations indicating opposite functions of cytokinins in the regulation of shoot and root meristem activity. Plant Cell 2003; 15: 2532–2550. 14555694 10.1105/tpc.014928 PMC280559

[bib72] Yang S, Yu H, Xu Y, Goh CJ. Investigation of cytokinin-deficient phenotypes in *Arabidopsis *by ectopic expression of orchid DSCKX1. FEBS Lett 2003; 555: 291–296. 14644430 10.1016/s0014-5793(03)01259-6

[bib73] Werner T, Holst K, Pörs Y et al. Cytokinin deficiency causes distinct changes of sink and source parameters in tobacco shoots and roots. J Exp Bot 2008; 59: 2659–2672. 18515826 10.1093/jxb/ern134 PMC2486470

[bib74] Eviatar-Ribak T, Shalit-Kaneh A, Chappell-Maor L, Amsellem Z, Eshed Y, Lifschitz E. A cytokinin-activating enzyme promotes tuber formation in tomato. Curr Biol 2013; 23: 1057–1064. 23746638 10.1016/j.cub.2013.04.061

[bib75] Moore B, Zhou L, Rolland F et al. Role of the *Arabidopsi*s glucose sensor HXK1 in nutrient, light, and hormonal signaling. Science 2003; 300: 332–336. 12690200 10.1126/science.1080585

[bib76] Arenas-Huertero F, Arroyo A, Zhou L, Sheen J, León P. Analysis of *Arabidopsis* glucose insensitive mutants, gin5 and gin6, reveals a central role of the plant hormone ABA in the regulation of plant vegetative development by sugar. Genes Dev 2000; 14: 2085–2096. 10950871 PMC316855

[bib77] Rook F, Corke F, Card R, Munz G, Smith C, Bevan MW. Impaired sucrose-induction mutants reveal the modulation of sugar-induced starch biosynthetic gene expression by abscisic acid signalling. Plant J 2001; 26: 421–433. 11439129 10.1046/j.1365-313x.2001.2641043.x

[bib78] Cheng W-H, Endo A, Zhou L et al. A unique short-chain dehydrogenase/reductase in arabidopsis glucose signaling and abscisic acid biosynthesis and functions. Plant Cell 2002; 14: 2723–2743. 12417697 10.1105/tpc.006494 PMC152723

[bib79] Ljung K, Nemhauser JL, Perata P. New mechanistic links between sugar and hormone signalling networks. Curr Opin Plant Biol 2015; 25: 130–137. 26037392 10.1016/j.pbi.2015.05.022

[bib80] Wind JJ, Peviani A, Snel B, Hanson J, Smeekens SC. ABI4: versatile activator and repressor. Trends Plant Sci 2013; 18: 125–132. 23182343 10.1016/j.tplants.2012.10.004

[bib81] Li P, Zhou H, Shi X et al. The ABI4-induced *Arabidopsis* ANAC060 transcription factor attenuates ABA signaling and renders seedlings sugar insensitive when present in the nucleus. PLoS Genet 2014; 10: e1004213. 24625790 10.1371/journal.pgen.1004213 PMC3953025

[bib82] Dominguez PG, Frankel N, Mazuch J et al. ASR1 mediates glucose-hormone cross talk by affecting sugar trafficking in tobacco plants. Plant Physiol 2013; 161: 1486–1500. 23302128 10.1104/pp.112.208199 PMC3585611

[bib83] LeClere S, Schmelz EA, Chourey PS. Sugar levels regulate tryptophan-dependent auxin biosynthesis in developing maize kernels. Plant Physiol 2010; 153: 306–318. 20237017 10.1104/pp.110.155226 PMC2862422

[bib84] Lilley JLS, Gee CW, Sairanen I, Ljung K, Nemhauser JL. An endogenous carbon-sensing pathway triggers increased auxin flux and hypocotyl elongation. Plant Physiol 2012; 160: 2261–2270. 23073695 10.1104/pp.112.205575 PMC3510146

[bib85] Sairanen I, Novák O, Pěnčík A et al. Soluble carbohydrates regulate auxin biosynthesis via PIF proteins in* Arabidopsis*. Plant Cell 2012; 24: 4907–4916. 23209113 10.1105/tpc.112.104794 PMC3556965

[bib86] Procko C, Crenshaw CM, Ljung K, Noel JP, Chory J. Cotyledon-generated auxin is required for shade-induced hypocotyl growth in *Brassica rapa*. Plant Physiol 2014; 165: 1285–1301. 24891610 10.1104/pp.114.241844 PMC4081337

[bib87] Hornitschek P, Kohnen MV, Lorrain S et al. Phytochrome interacting factors 4 and 5 control seedling growth in changing light conditions by directly controlling auxin signaling. Plant J 2012; 71: 699–711. 22536829 10.1111/j.1365-313X.2012.05033.x

[bib88] Mishra BS, Singh M, Aggrawal P, Laxmi A. Glucose and auxin signaling interaction in controlling *Arabidopsis thaliana *seedlings root growth and development. PLoS ONE 2009; 4: e4502. 19223973 10.1371/journal.pone.0004502 PMC2637607

[bib89] Kushwah S, Laxmi A. The interaction between glucose and cytokinin signal transduction pathway in Arabidopsis thaliana. Plant Cell Environ 2014; 37: 235–253. 23763631 10.1111/pce.12149

[bib90] Li G, Ma J, Tan M et al. Transcriptome analysis reveals the effects of sugar metabolism and auxin and cytokinin signaling pathways on root growth and development of grafted apple. BMC Genomics 2016; 17: 1–17. 26923909 10.1186/s12864-016-2484-x PMC4770530

