# Supplementary figures and images for: Apple dwarfing rootstocks exhibit an imbalance in carbohydrate allocation and reduced cell growth and metabolism
Source: Hortic Res. 2017 Apr 5;4:17009–. doi: 10.1038/hortres.2017.9 (PMC5381684; doi:10.1038/hortres.2017.9)

## Slide 1
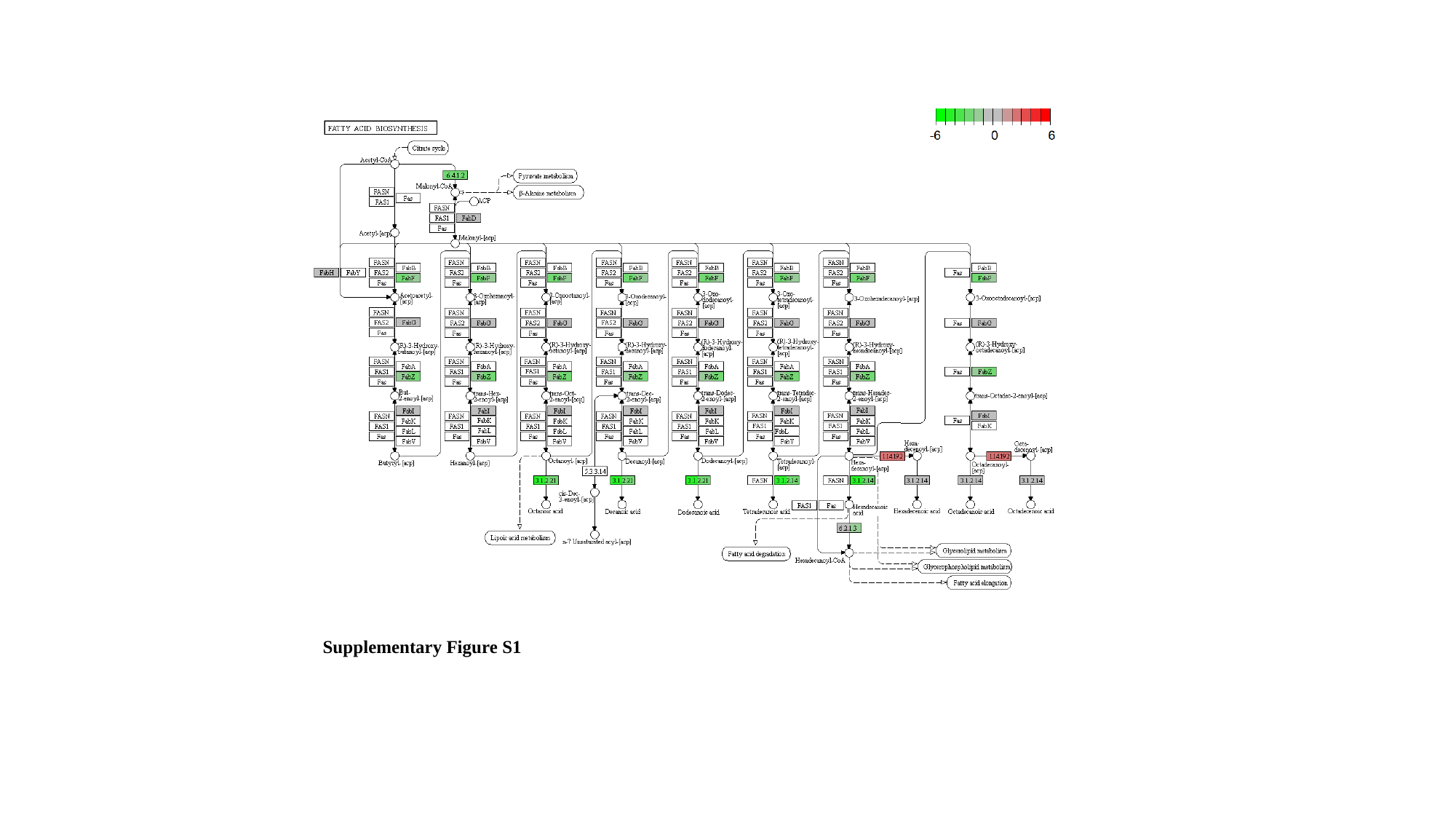

Supplementary Figure S1

Supplement: Supplementary Figure S1 [file hortres20179-s2.pptx]

## Slide 1
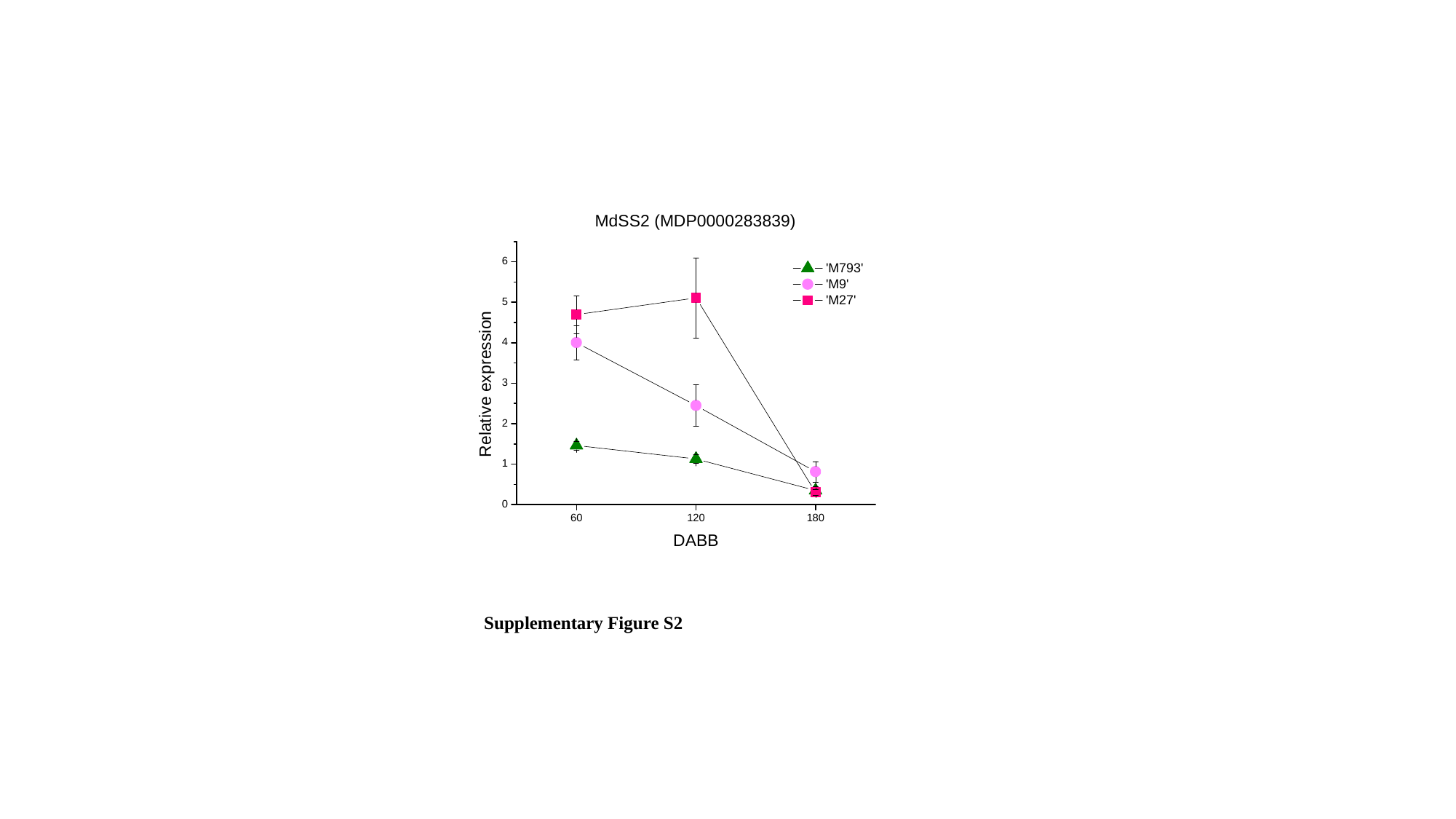

Supplementary Figure S2

Supplement: Supplementary Figure S2 [file hortres20179-s3.pptx]

## Slide 1
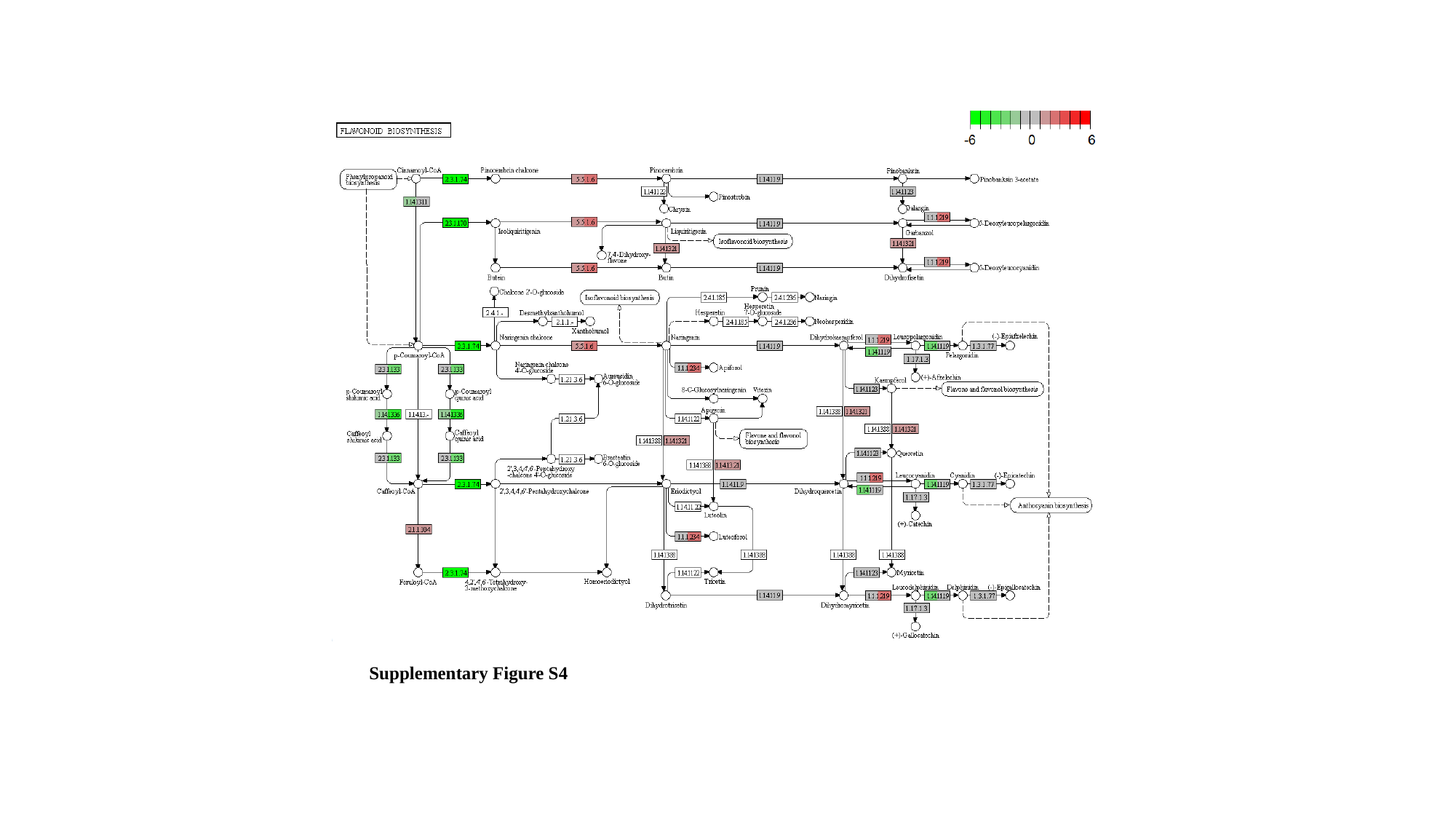

Supplementary Figure S4

Supplement: Supplementary Figure S4 [file hortres20179-s5.pptx]

## Slide 1
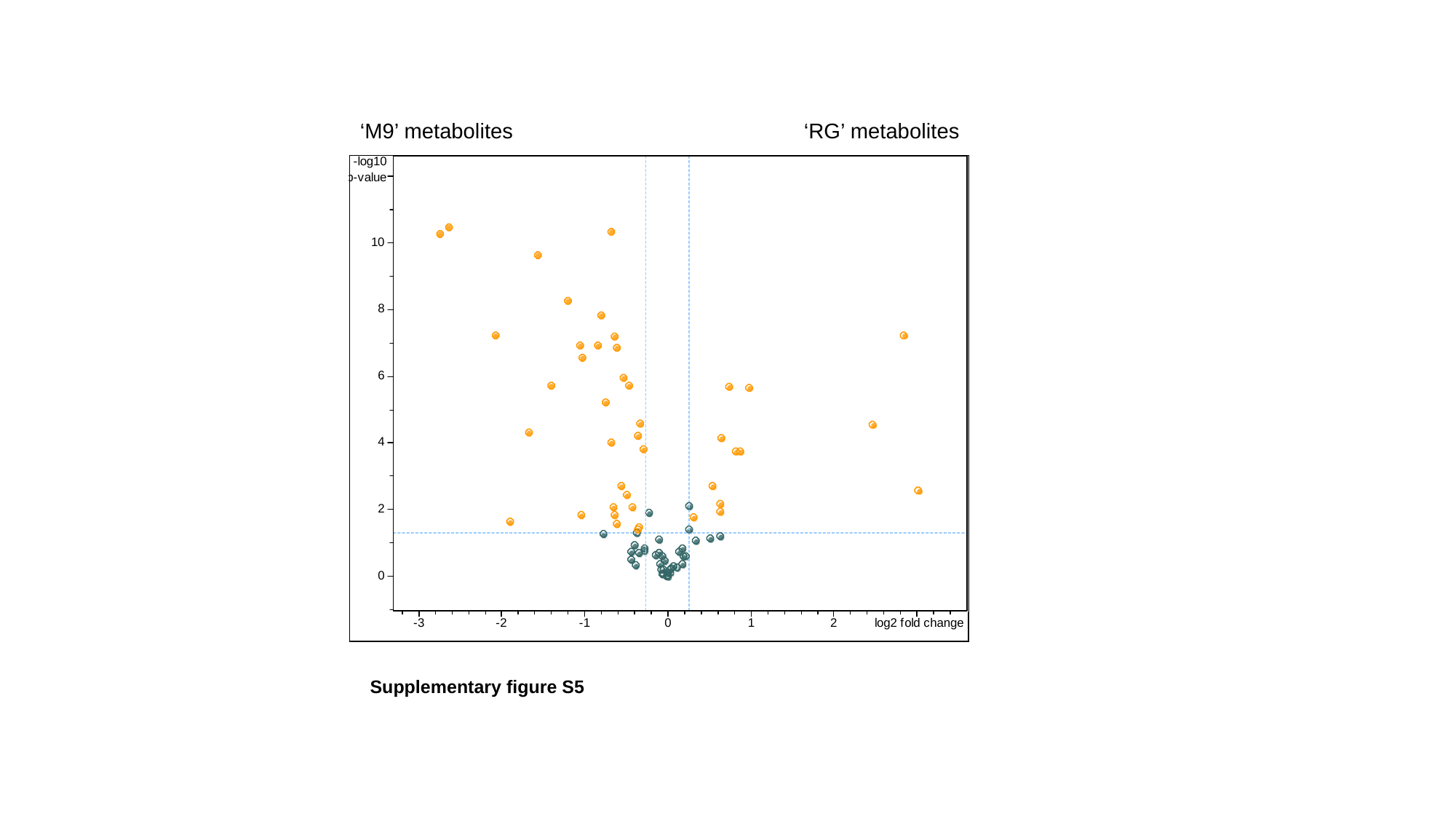

‘M9’ metabolites
‘RG’ metabolites
Supplementary figure S5

Supplement: Supplementary Figure S5 [file hortres20179-s6.pptx]

## Slide 1
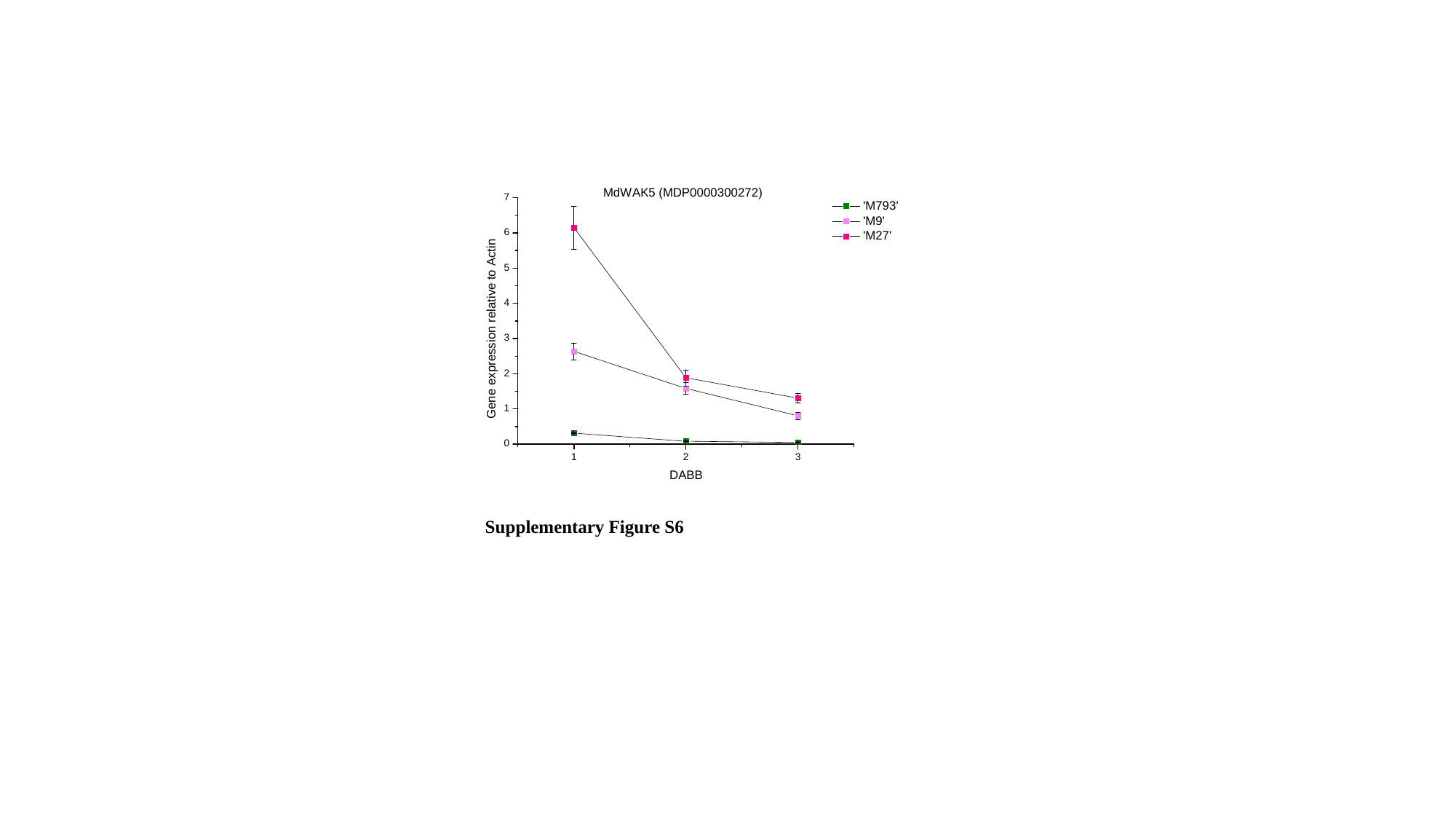

Supplementary Figure S6

Supplement: Supplementary Figure S6 [file hortres20179-s7.pptx]
