# Supplementary material for: Apple dwarfing rootstocks exhibit an imbalance in carbohydrate allocation and reduced cell growth and metabolism
Source: Hortic Res. 2017 Apr 5;4:17009–. doi: 10.1038/hortres.2017.9 (PMC5381684; doi:10.1038/hortres.2017.9)
Supplement: Supplementary Figure S3 [file hortres20179-s4.pptx]

## Slide 1
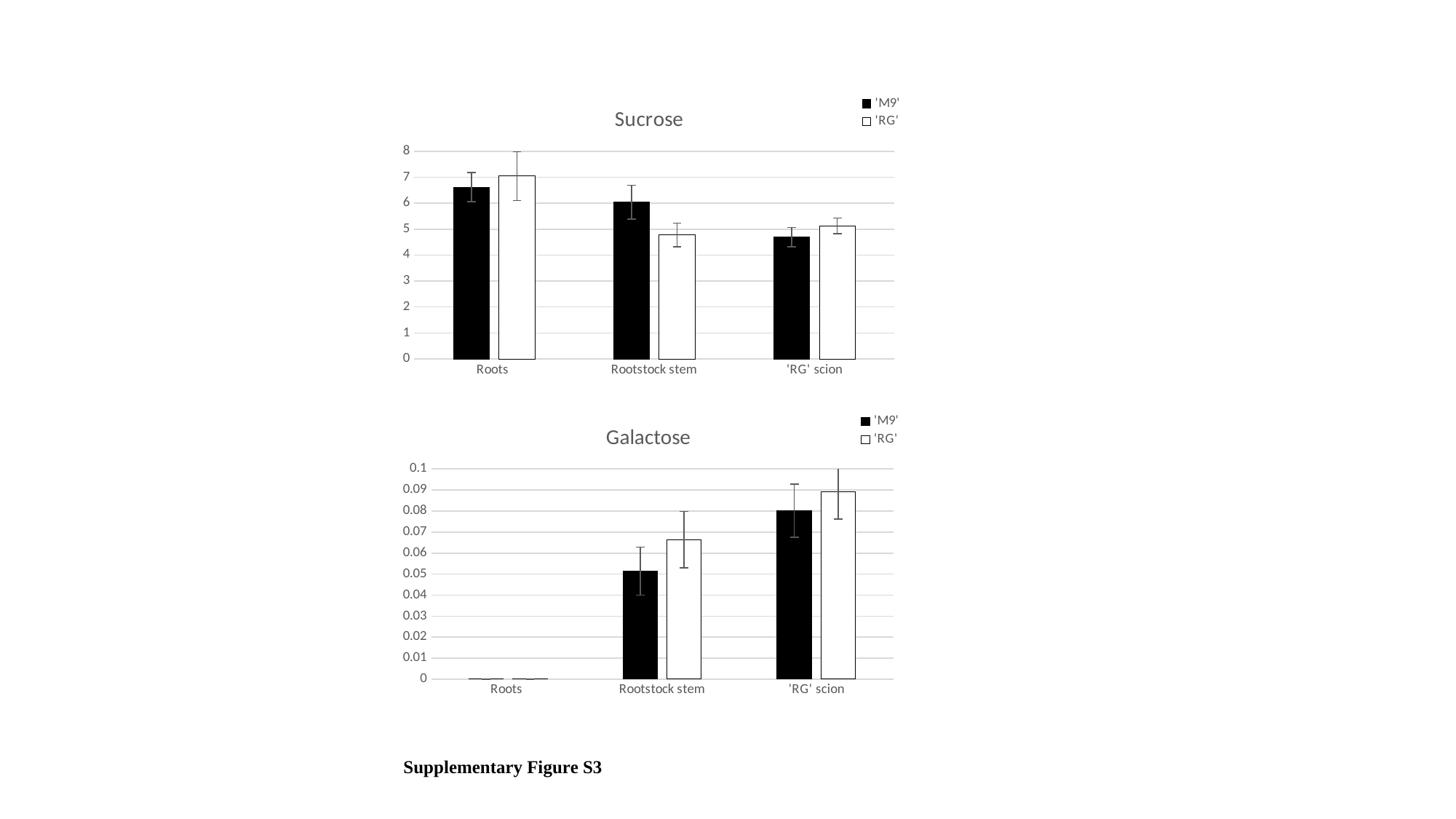

### Chart: Sucrose
| Category | | |
|---|---|---|
| Roots | 6.617563934650381 | 7.042282615248852 |
| Rootstock stem | 6.038109878338445 | 4.776706818372619 |
| 'RG' scion | 4.692614784469193 | 5.125623903654435 |
### Chart: Galactose
| Category | 'M9' | 'RG' |
|---|---|---|
| Roots | 0.0 | 0.0 |
| Rootstock stem | 0.0514236640863466 | 0.06641799167286072 |
| 'RG' scion | 0.08013729113294481 | 0.08916466454348206 |Supplementary Figure S3
